# 血友病合并抑制物诊断与治疗中国指南（2023年版）

**DOI:** 10.3760/cma.j.issn.0253-2727.2023.11.001

**Published:** 2023-11

**Authors:** 中华医学会血液学分会血栓与止血学组 中国血友病协作组

一、概述

抑制物是血友病患者接受外源性凝血因子Ⅷ/Ⅸ（FⅧ/FⅨ）输注后产生的抗FⅧ/FⅨ同种中和抗体。抑制物是血友病治疗过程中最严重、最棘手的并发症。中华医学会血液学分会血栓与止血学组、中国血友病协作组于2018年制订了《凝血因子Ⅷ/Ⅸ抑制物诊断与治疗中国指南》[1]。此后，又分别对国内同行和血友病患者进行了抑制物诊治现状的专项调查，结果表明有关人员对于血友病合并抑制物的认知水平有了较大提高，也有不尽如人意之处[2]–[4]。近年的研究揭示了血友病合并抑制物的发病机制，同时新药的不断涌现也为抑制物患者出血的预防及治疗提供了更多的选择[5]–[8]。为进一步提高对血友病合并抑制物的认识，作到发现及时、处理规范，特制订此指南供国内同行参考。

二、基本概念

详见《凝血因子Ⅷ/Ⅸ抑制物诊断与治疗中国指南（2018版）》[1]。

三、推荐等级

根据GRADE方法[9]，本指南推荐等级如下：

1级推荐：相当于“指南推荐”，代表该推荐对患者的安全性及获益明显高于风险和负担。1B级：该推荐至少有一项观察性或干预性研究的数据支持，且该推荐在大多数情况下适用于大多数患者；1C级：该推荐缺乏此类证据支持，但是仍然对患者的安全或获益很重要。

2级推荐：相当于“指南建议”，用于表示较弱的推荐，该建议可能会随着更新证据的出现发生改变。2B级：病例登记或研究数据支持该建议；2C级：无前述数据支持。

四、FⅧ/FⅨ抑制物（同种抗体）的危险因素

抑制物发生的危险因素包括遗传因素和非遗传因素。

遗传因素主要有疾病严重程度[10]–[11]、种族和家族史[12]–[13]、基因突变类型[10],[13]–[14]等。F8基因突变类型是最重要的抑制物产生危险因素[14]。与重型血友病A（HA）患者产生抑制物密切相关的主要突变类型包括大片段缺失、无义突变、22号内含子倒位，其次为小片段缺失和插入、错义突变等。不同类型的基因突变导致抑制物产生的风险差异，可能与体内存在FⅧ抗原量有关。SIPPET研究发现，部分无效突变（如大片段缺失）的患者体内仍然能够检测到微量FⅧ抗原，总体来说体内不存在FⅧ抗原的患者抑制物风险较抗原阳性患者增加3.5倍[15]。有大片段基因缺失或无效突变的血友病B（HB）患者抑制物发生率高[2],[16]，文献报道分别为33.3％、26.9％[16]。

非遗传因素包括治疗方式[10],[17]、凝血因子输注剂量[10],[17]、首次治疗年龄[17]、高强度因子治疗[10],[17]、凝血因子产品种类[18]–[24]、暴露天数（exposure day, ED）等。按需治疗与预防治疗的抑制物风险不同，RODIN研究发现，预防治疗的患者在20 ED之后抑制物风险显著降低[17]。FⅧ浓缩物类型是最有争议的危险因素之一。SIPPET研究结果提示，基因重组人FⅧ（rhFⅧ）抑制物风险是含血管性血友病因子（vWF）分子的血源性FⅧ（plasma-derived FⅧ，pdFⅧ）的1.87倍[23]。然而，许多临床研究和Meta分析结果相互矛盾[20]–[22],[25]。关于高强度因子治疗，CANAL研究表明，初次接触FⅧ超过5 d的患者抑制物产生风险较1～3 d的患者增加3.2倍[10]。RODIN研究表明首次接触FⅧ超过3～5 d的患者抑制物产生风险增加1.4倍，首次接触FⅧ 5～10 d或超过10 d的患者抑制物产生风险增加2倍[17]。手术增加抑制物产生的风险与高强度因子治疗以及手术过程中炎症反应有关。疫苗接种曾被认为是抑制物产生的危险因素，但目前的研究并未发现疫苗可导致抑制物风险增高[26]–[27]。重型HA患者抑制物发生率为25％～35％[28]，产生抑制物的患者中36％的FⅧ抑制物发生在前10 ED，79％发生在前20 ED，96％发生在前50 ED，在75 ED后很少有新的抑制物产生[28]。轻/中型血友病患者抑制物发生率随着ED增加而逐渐增高，在50 ED时抑制物的发生率为6.7％，在75 ED后仍然有抑制物产生，到100 ED时为13.3％[1]。HB患者抑制物主要产生于前20 ED，且往往出现在2岁之前。来自PedNet研究发现，HB发生抑制物的中位ED为11（IQR 6.5～36.5）d，75 ED时抑制物发生率为9.3％[16]。

**推荐意见** 遗传因素是血友病患者抑制物产生最重要的危险因素，建议条件允许的患者都要进行F8或F9基因检测（1B）。

**推荐意见** 对于因为病情需要的初治重型患儿，使用凝血因子产品前应与患儿父母或者监护人进行充分沟通，以便其知晓抑制物发生的风险（1B）。

**推荐意见** 条件允许时，重型血友病患者前75 ED内尽量避免手术以及高强度凝血因子输注，尤其是连续5 d以上输注凝血因子（1B）。

五、抑制物的筛查

若有以下情况，应及时进行抑制物检测：①凝血因子替代治疗效果不如既往；②在规范预防治疗情况下，出血频率增加或者仍有靶关节出血；③高强度输注凝血因子后，如连续输注≥5 d；④手术前；⑤手术后凝血因子替代治疗疗效不佳；⑥对FⅨ制剂过敏的HB患者；⑦轻/中型血友病患者出血表现加重（如出现严重的自发关节和肌肉出血）。

**推荐意见** 重型血友病患者在开始进行凝血因子治疗时应该加强抑制物的筛查，以便及早发现。对于儿童患者，建议在首次接受凝血因子输注后的前20 ED每5 ED检测1次，在21～50 ED内每10 ED检测1次，此后每年至少检测2次，直至150 ED（1B）。

六、抑制物的实验室检测

1. 活化部分凝血活酶时间（APTT）混合血浆纠正试验：为了鉴别APTT延长是由于凝血因子缺乏或抑制物存在，可进行APTT混合血浆纠正试验。将正常人混合血浆和患者血浆1∶1混合后，分别于即刻和37 °C孵育2 h后测定APTT，并与正常人混合血浆和患者血浆的APTT进行比较（图1），若患者的APTT延长不能被正常人混合血浆纠正，提示可能存在抑制物。由于试验的复杂性及缺乏标准化，不同实验室对于纠正的判定并无统一标准，常用几种方法包括正常参考区间法、循环抗凝物指数方法、百分比纠正法或延长百分比法等[29]，各实验室应根据自身经验建立合适的判定标准。FⅧ抑制物具有时间及温度依赖性，多数抗体表现为混合孵育2 h后APTT较即刻混合明显延长（延长3 s以上或超过10％～15％），但需要指出的是：少部分高滴度FⅧ抑制物具有即刻反应的特点，即刻混合后APTT不能纠正，而部分低滴度FⅧ抑制物（较弱抗体存在时）可能表现为患者的APTT延长被完全纠正，时间温度依赖性不明显；也有少部分（10％～15％）狼疮抗凝物可表现为时间依赖性。因此，APTT纠正试验可作为抑制物的初筛检查，但确诊抑制物还需结合狼疮抗凝物、抑制物定量检测以及FⅧ回收率综合判断。

**图1:**
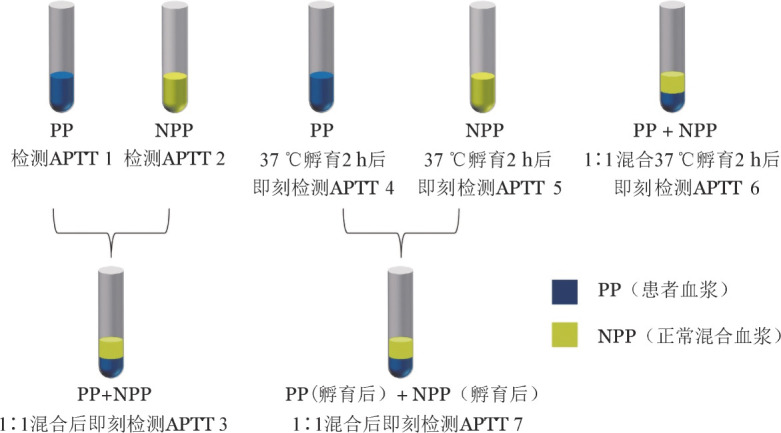
活化部分凝血活酶时间（APTT）纠正试验示意图

2. 抑制物定量检测（以FⅧ抑制物为例）：Bethesda法和Nijmegen改良法用于确诊抑制物，将不同稀释度的患者血浆与正常人血浆1∶1混合，37 °C孵育2 h，测定剩余FⅧ活性（FⅧ∶C），具体操作步骤见图2，中和正常人血浆50％ FⅧ∶C时抑制物的滴度定义为1个Bethesda单位（BU），1～4周内连续两次用Bethesda法或Nijmegen改良法检测患者抑制物滴度≥0.6 BU/ml为阳性。相较Bethesda法，Nijmegen法进行了以下改良：①正常对照血浆用咪唑缓冲液调整PH至7.4；②乏FⅧ血浆作为患者血浆和对照血浆的稀释液，可以增加滴度<2 BU/ml抑制物检测的特异性和灵敏性。没有经过足够洗脱期或者凝血因子活性>5 IU/dl的血浆样本，为避免患者体内残余FⅧ对检测的干扰[30]–[31]，可在56 °C下孵育30 min以灭活血浆中FⅧ，再进行抑制物定量检测。血浆中存在肝素、狼疮抗凝物等非特异性抗凝物质和某些药物时影响基于凝固法检测凝血因子抑制物的检测结果，可选用基于发色底物法凝血因子抑制物检测（chromogenic Bethesda assay, CBA）[32]。需要注意的是Bethesda法检测的是中和性抗体，小部分非中和性FⅧ抗体（加快FⅧ清除，缩短半衰期），Bethesda法无法检出。对使用猪rFⅧ治疗的血友病患者，除需检测抗人FⅧ抑制物外还需定期检测抗猪rFⅧ抑制物。

**图2:**
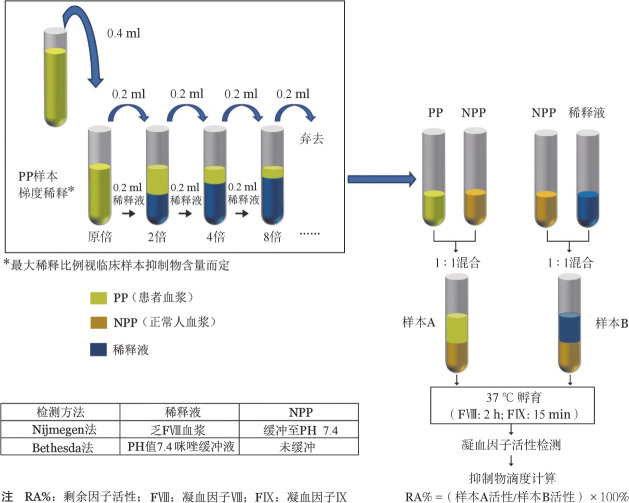
Bethesda法和Nijmegen法检测凝血因子抑制物

**推荐意见** FⅧ抑制物<2.0 BU/ml、FⅨ抑制物<1.0 BU/ml，为抑制物检测“灰区”[33]，可采用回收试验进一步确认FⅧ/FⅨ抑制物存在；同时重复Bethesda法、Nijmegen改良法检测或者CBA检测进行抑制物定量测定（1B）。

3. 狼疮抗凝物检测：狼疮抗凝物可引起APTT延长，不能被正常人血浆纠正，虽然狼疮抗凝物多数表现为即时作用抗体，但有10％～15％狼疮抗凝物具有时间依赖性，不能完全依靠时间依赖性特点来区分FⅧ抑制物和狼疮抗凝物，同时狼疮抗凝物的存在会干扰凝血因子活性和抑制物定量检测，导致内源性凝血因子活性减低和凝血因子抑制物假阳性。狼疮抗凝物检测包括稀释的蝰蛇毒试验（dRVVT）和对狼疮抗凝物敏感的APTT检测（silica clotting time, SCT），其原理是利用补充过量磷脂能缩短或纠正抗磷脂抗体引起的凝固时间[34]延长。

**推荐意见** 由于狼疮抗凝物存在异质性，建议结合两种不同方法学进行检测，首选dRVVT检测，其次可以选择对狼疮抗凝物敏感的APTT（含少量磷脂，以硅作为激活剂）检测（1B）。

**推荐意见** 如存在多个内源性凝血因子活性减低时，需排除狼疮抗凝物以及FⅧ或者FⅨ高滴度抑制物的影响（1B）。

4. 新型非因子药物治疗实验室监测[35]：非因子治疗药物的问世，对血友病患者的实验室监测提出新的挑战。目前可用于血友病患者的非因子治疗药物包括FⅧ模拟制剂艾美赛珠单抗及凝血再平衡制剂［如降低抗凝血酶（ATⅢ）表达的小干扰RNA（siRNA-ATⅢ）］和组织因子途径抑制剂（tissue factor pathway inhibitor, TFPi）的抗体（anti-TFPi）。使用艾美赛珠单抗会明显缩短APTT，使其在正常范围或低于正常范围，因此也会影响基于APTT凝固法的FⅧ∶C和抑制物检测，建议检测FⅧ抑制物滴度时使用牛源性发色底物法试剂进行，而判断艾美赛珠单抗的疗效时需要用人源性发色底物法试剂来进行FⅧ∶C检测。对于siRNA-ATⅢ，患者肝细胞中的残余寡核苷酸水平无法实际测量，但可以检测血浆中残存的ATⅢ抗原水平和活性水平。目前还没有明确的检测方法来测量anti-TFPi药物浓度，可选择检测TFPi的抗原水平和依赖组织因子的发色底物法检测TFPi活性[36]。非因子药物使用后对患者凝血潜能的影响是实验室监测的重点。非因子药物治疗前后可进行凝血酶生成试验（thrombin generation assay, TGA）、血栓弹力图及血块波形分析（clot waveform analysis, CWA），通过观察患者止血潜能变化来分析其疗效[37]。但需要注意这些检测并不能提供有关药物的真正FⅧ等效浓度。

七、HA合并FⅧ抑制物的处理

（一）出血治疗

抑制物患者出血的治疗方案取决于抑制物滴度、出血部位/严重程度、药物可及性以及疗效。

1. 大剂量FⅧ治疗：对于低滴度低反应抑制物，加大剂量的FⅧ替代治疗仍然可能有效。对于低滴度高反应的患者来说，在某些危及生命的紧急情况下（图3），仍然可以采取大剂量输注FⅧ的办法，虽然有可能导致抑制物滴度反应性增高的风险，但大剂量FⅧ输注对急性出血（特别是出血量较大时）仍可能有效。所需要FⅧ的量包括用来中和抑制物的量以及止血所需要的量。用来中和抑制物的FⅧ用量算法如下：中和抑制物所需因子量+达预期因子水平所需因子量=［抑制物滴度（BU/ml）×20（IU）×体重（kg）］+［预期因子水平×体重（kg）/2］。如果大剂量FⅧ治疗后止血疗效不佳或者抑制物升高至≥5 BU/ml，改为旁路途径止血药物。

**图3:**
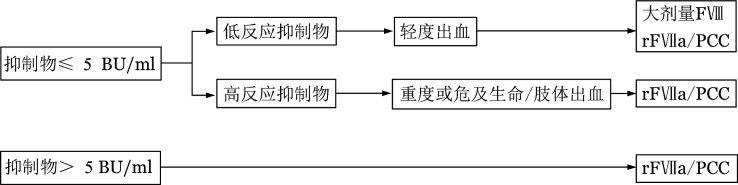
血友病A合并凝血因子Ⅷ（FⅧ）抑制物的出血治疗选择 rFⅦa：基因重组活化凝血因子Ⅶ；PCC：凝血酶原复合物

2. 旁路制剂治疗：对于高滴度抑制物（≥5 BU/ml）的患者，低滴度抑制物大剂量FⅧ治疗疗效不好或者达不到有效FⅧ∶C的患者，建议换用旁路制剂治疗[31]。可供选择的“旁路途径”药物包括基因重组活化凝血因子Ⅶ（rFⅦa）及活化凝血酶原复合物（aPCC），国内无aPCC产品，所以用PCC代替。文献报道治疗关节出血时，rFⅦa与aPCC的疗效类似，为80％～90％[38]。在治疗关节出血时，2剂rFⅦa 90～120 µg/kg与1剂aPCC 75～100 IU/kg疗效相当[38]。

rFⅦa用法为90 µg/kg每2～4 h 1次静脉注射。有学者认为，由于aPCC/PCC含有微量的FⅧ，在30％的患者可能引起抑制物反应性增高，因此，在治疗出血时选择rFⅦa可能更好。

PCC的用法为50～100 IU/kg每8～12 h 1次静脉给药，一天总量不超过150 IU/kg。对于一般的关节出血，PCC用量可为每次50～75 IU/kg，严重或者危及生命出血，可增至每次100 IU/kg。既往发生过动静脉血栓的患者，可适当或酌情调整剂量。PCC应尽量避免与抗纤溶药物同时使用，如必须使用建议间隔6 h以上，以降低血栓事件风险。

对单一旁路途径无效或费用有限者，可采用PCC与rFⅦa序贯疗法，给药剂量在单一旁路途径给药基础上根据患者情况调整。两种药物联用时应严密监测血栓相关的症状以及实验室指标，以防止发生血栓。

HA合并FⅧ抑制物患者的出血治疗选择见图3。

**推荐意见** 低滴度低反应抑制物患者或者高反应抑制物患者在抑制物滴度<5 BU/ml时，如发生危及生命的出血，可选择加大剂量的FⅧ治疗（1C）。用药期间可监测FⅧ∶C和APTT，以调整治疗剂量，在抑制物滴度升高至≥5 BU/ml或止血疗效欠佳时再选择旁路制剂。也可直接选择旁路制剂，其中首选rFⅦa（1C）。

**推荐意见** 高滴度抑制物患者在发生出血时，建议直接选择旁路制剂，其中首选rFⅦa治疗（1C）。

**推荐意见** 单一旁路制剂无效，或者费用有限时，可采取PCC与rFⅦa序贯疗法，联用两种药物时应严密监测血栓事件的发生（1C）。

（二）艾美赛珠单抗预防治疗

艾美赛珠单抗是一种双特异性的单克隆抗体，通过模拟FⅧa的辅因子功能，可同时桥接FⅨa和FⅩ，使FⅩ在没有FⅧ的情况下得以继续激活，重新恢复凝血瀑布反应。由于艾美赛珠单抗的药代动力学特征，不适合用作按需治疗。目前该药物在国内已获得批准用于HA合并抑制物患者的常规预防治疗。相比旁路制剂按需或预防治疗，艾美赛珠单抗预防治疗在控制出血、恢复靶关节、提高血友病患者生活质量方面更有优势。

**推荐意见** 艾美赛珠单抗适用于HA伴抑制物的预防治疗，不适用于按需治疗（1B）。

1. 艾美赛珠单抗的用法：在用药上，前4周需要给予负荷剂量3 mg/kg每周1次皮下注射，以快速达到目标血药浓度，第5周起给予维持剂量1.5 mg/kg每周1次，或3 mg/kg每2周1次，也可以6 mg/kg每4周1次。一些临床医师也在探索小剂量的方式。一项回顾性研究纳入13例低剂量使用艾美塞珠单抗的HA儿童（包括9例抑制物患者），患者出血得到明显的改善[39]。

**推荐意见** 条件允许时建议采用标准剂量艾美赛珠单抗治疗；在经济状况不允许时，可尝试小剂量艾美赛珠单抗治疗，但具体剂量目前尚不明确（2B）。

2. 艾美赛珠单抗与凝血因子产品的合用：对于严重或者致命性的出血仍需要用到旁路制剂止血。在HAVEN 1研究中，3例患者在使用了大剂量的aPCC后发生血栓性微血管病事件[40]。因此在使用艾美赛珠单抗期间应尽量避免使用aPCC/ PCC，首选rFⅦa治疗，初始剂量应≤90 µg/kg，重复给药时，治疗间隔应>2 h，可45 µg/kg每4 h 1次，如果疗效不佳，剂量可增至90～120 µg/kg每2～4 h 1次，直至出血控制。低滴度抑制物患者可选择FⅧ治疗；对于合并血栓风险因素（肥胖、深静脉血栓、吸烟、炎症等）的患者，应用rFⅦa应非常小心，因为有发生非ST段抬高心肌梗死或者肺栓塞的风险。高滴度抑制物如果rFⅦa疗效不佳或者药物获取困难，必要时可考虑低剂量PCC治疗，建议单次PCC剂量<50 IU/kg（如25～50 IU/kg），但在使用PCC期间，需要密切警惕血栓事件发生。如果患者需要多次给予PCC治疗，每日总量<100 IU/kg。艾美赛珠单抗需在专业医师的指导下使用[41]。

**推荐意见** 艾美赛珠单抗在旁路制剂合用时，首选rFⅦa治疗，无效或者实际情况不允许时，必要时可考虑低剂量PCC，用药期间密切监测血栓形成的风险（1B）。

（三）旁路制剂的预防治疗

目前旁路制剂在国内并未获批用于血友病伴抑制物的预防治疗。国外数据显示，rFⅦa可减少抑制物患者的出血[42]，但由于rFⅦa半衰期短，且费用较高，并未广泛在患者中使用。目前国内没有PCC在抑制物患者中预防治疗的研究报道。条件允许时可考虑rFⅦa或者PCC预防治疗。

（四）其他药物的治疗

可以恢复凝血平衡的其他非因子类药物如anti-TFPi以及anti-ATⅢ，在HA和HB伴抑制物患者的预防治疗中已取得良好的疗效[7]–[8],[43]。这些新的药物主要以皮下注射的方式给药，操作相对容易，也避免了静脉穿刺的痛苦。从蛇毒中提取的国产FⅩ激活剂（STSP-0601）在HA和HB抑制物患者的Ⅰa/Ⅰb临床试验中显示可显著缩短患者APTT并改善凝血酶产生的相关试验标[44]。Sevenfact是FDA批准的第二个rFⅦa产品，获批用于12岁以上HA、HB伴抑制物患者出血的治疗。目前在小于12岁HA或者HA伴抑制物患者中的Ⅲ期临床研究也显示较好的止血疗效[45]。在HA伴抑制物的Ⅱ期临床试验中，单次输注猪rFⅧ后，患者出血即可得到改善[46]。这些新药为患者的治疗带来新的选择。

（五）免疫耐受诱导治疗（ITI）

ITI是目前主要的清除重型血友病抑制物的治疗方案。ITI治疗是指定期反复输注凝血因子，使得抑制物患者免疫系统对外源性凝血因子产生耐受，从而达到清除抑制物的目的。ITI治疗建立免疫耐受的确切机制尚不明确，可能的机制包括诱导抗原特异性记忆B细胞的凋亡、诱导抗原特异性效应T细胞的无能以及诱导调节性T细胞的产生，同时可诱导抗抑制物特异性抗体的形成[47]。HA伴抑制物患者的ITI总体有效率约为70％[48]–[49]。

1. ITI疗效的预测[50]：目前认为以下患者ITI疗效可能较好：①开始ITI之前抑制物滴度<10 BU/ml；②抑制物滴度历史峰值<200 BU/ml；③ITI期间抑制物滴度峰值<100 BU/ml；④从诊断到开始ITI的时间<5年；⑤ITI开始后没有间断。而有如下指标的患者ITI疗效可能较差：①开始ITI之前抑制物滴度≥10 BU/ml；②抑制物滴度历史峰值≥200 BU/ml；③ITI期间抑制物滴度峰值≥100 BU/ml；④从诊断到开始ITI的时间≥5年；⑤ITI开始后间断≥2周。

2. ITI的适用人群以及治疗时机：所有抑制物持续阳性重型HA患者均应立即接受ITI。如需推迟ITI，建议等待时间不宜过长，尽量不超过1年[31]。

3. ITI使用的制剂：研究发现，在使用rhFⅧ后发生抑制物的患者中，用含有vWF的血源性FⅧ进行ITI具有较高的成功率[51]–[52]。但文献荟萃分析结果显示血源性FⅧ和rhFⅧ的ITI成功率并无差别[11]。

4. ITI的剂量选择：关于ITI剂量的选择，目前没有定论。但有学者认为，对于预后不良或者高滴度高反应抑制物的患者，大剂量ITI方案疗效更好，而对于良好预后患者，或者低滴度低反应抑制物患者倾向用于中小剂量方案[53]。为了比较大剂量（200 U·kg^−1^·d^−1^）和低剂量（50 U/kg每周3次）方案的优劣，国际ITI研究组对历史抑制物滴度高峰小于200 BU/ml的预后良好患者进行了随机对照研究，虽然由于在ITI成功前低剂量组出血更频繁而提前终止了该试验，但两组的ITI成功率没有显著差别（69.7％），当然低剂量组获得成功所需时间更长[48]。然而在土耳其的两项研究中，对于高滴度抑制物患者采用小剂量ITI并未取得良好疗效：在其中一项研究中，26.3％的患者成功清除了抑制物[54]；在另一项研究中，33.3％的患者清除了抑制物，同时FⅧ回收率恢复到66％[55]。Li等[56]在高滴度抑制物患者（ITI开始时抑制物滴度≥10 BU/ml）中采用小剂量ITI治疗方案（50 IU/kg隔日1次），其中对于历史抑制物滴度高（≥100 BU/ml）以及ITI开始时抑制物滴度高（≥40 BU/ml）或治疗过程中抑制物滴度增高（≥40 BU/ml）或ITI治疗前3个月滴度下降不理想患者的联合免疫抑制治疗。该研究发现，合并不良ITI预后因素患者的总体疗效与国际ITI研究组中合并良好预后因素患者类似，且疗效高于土耳其ITI疗效。提示免疫抑制治疗可提高低剂量ITI治疗预后不良患者的疗效。然而，该研究也发现，抑制物峰值滴度大于100 BU/ml患者的转阴时间以及FⅧ回收率恢复时间长于其他患者。在该研究团队前期的临床研究中，6例历史抑制物峰值>200 BU/ml的患者只有1例清除了抑制物[57]。因此对于抑制物高的患者（≥200 BU/ml），建议采用更大剂量的方案，必要时可加用免疫抑制治疗。

5. ITI疗效评估：ITI疗效评估标准见表1。

**表1:** 免疫耐受诱导治疗（ITI）疗效评估标准

疗效分类	疗效标准
完全耐受	抑制物持续阴性（<0.6 BU/ml），且FⅧ回收率≥66%及FⅧ（标准半衰期产品）半衰期≥6 h
部分耐受	抑制物滴度<5 BU/ml，虽然FⅧ回收率<66%和（或）半衰期<6 h，但是使用FⅧ可阻止出血
无效	不能达到完全和部分耐受。一般来说，在6个月内抑制物滴度下降不足20%，或者经过3~5年ITI后抑制物滴度仍>5 BU/ml提示ITI可能无效

注 FⅧ：凝血因子Ⅷ

**推荐意见** 抑制物阳性的患者无论是成人还是儿童，一旦确诊均应立即开始抑制物清除治疗（1B）。

**推荐意见** 可根据患者抑制物滴度，采取不同ITI剂量：抑制物滴度小于200 BU/ml的患者，可采取小剂量ITI治疗，比如25～50 IU/kg隔日1次；疗效不佳时可考虑联用免疫抑制剂，若抑制物滴度≥200 BU/ml，需采取中剂量（100 IU·kg^−1^·d^−1^）或大剂量ITI（200 IU·kg^−1^·d^−1^），必要时联用免疫抑制治疗（1B）。

**推荐意见** 一旦开始ITI，不宜随便中止，以免影响后续ITI的疗效（1C）。

6. 艾美赛珠单抗使用期间ITI的问题：ITI期间同时使用艾美赛珠单抗的经验较少。理论上，艾美赛珠单抗可以降低小剂量ITI治疗期间患者的出血风险，有利于小剂量ITI的实施。一项研究尝试使用“Atlanta”方案：患者先接受负荷剂量的艾美赛珠单抗治疗，第5周起给予维持剂量，至少4周后接受小剂量ITI治疗方案：rhFⅦ或者血源性FⅧ 50～100 IU/kg每周3次。入组7例患者，经过中位35（21～40）周的治疗，3例患者抑制物转阴，其余4例患者抑制物滴度也呈减低趋势[58]。

**推荐意见** 对于接受小剂量ITI且出血风险较高的患者，建议尝试联合艾美赛珠单抗治疗（2B）。

八、HB合并FⅨ抑制物的处理

FⅨ抑制物的产生往往伴随着过敏反应出现，因此在患者接受FⅨ替代治疗过程中，即使出现轻微过敏反应，也应进行抑制物筛查。此外，HB伴有抑制物患者开展ITI治疗，即使没有接受长期或大剂量FⅨ替代治疗，也有可能引起肾病综合征[59]–[60]。

关于HB伴抑制物的治疗选择，可采用以下方法：①针对抑制物患者出血时，可选择rFⅦa治疗。对于低滴度抑制物患者，若FⅨ替代治疗有效、无过敏反应，在rFⅦa不可及的情况下，可酌情选择PCC或大剂量FⅨ继续替代治疗，治疗过程需观察过敏以及肾病的风险。②HB伴抑制物患者可考虑选择rFⅦa预防治疗。剂量选择同HA伴抑制物患者。③高滴度抑制物、无FⅨ过敏反应的患者可酌情选择ITI。由于HB伴抑制物发生率低，目前尚无统一治疗方案，因有肾病的风险，建议小剂量ITI。HB伴抑制物患者治疗效果不及HA，总体有效率约为30％[31]，HB伴抑制物ITI治疗效果不佳，可选择联合应用免疫抑制剂（糖皮质激素、环孢素A、霉酚酸酯、利妥昔单抗等）[1]。④非因子类药物（anti-TFPi、siRNA-ATⅢ）可作为HB伴抑制物患者预防出血的新选择。

**推荐意见** 高滴度抑制物患者，建议首选免疫抑制药物清除抑制物；无过敏或肾病患者在充分与患者以及家属沟通后，可考虑采取小剂量ITI。治疗过程中注意肾病以及过敏的风险（1B）。

九、抑制物患者的手术问题

血友病患者抑制物阳性是手术止血管理的一大挑战。高滴度、高反应性抑制物患者应尽量避免手术。抑制物阳性患者必须手术时，需要评估医院是否具备血友病综合诊疗团队和血友病手术经验丰富的专业医师，是否能够快速动员检验、药品、血库等相关资源，以及患者与家属是否已经充分知情手术风险。
